# Metabolic changes after polytrauma: an imperative for early nutritional support

**DOI:** 10.1186/1749-7922-1-29

**Published:** 2006-10-04

**Authors:** Erik Hasenboehler, Allison Williams, Iris Leinhase, Steven J Morgan, Wade R Smith, Ernest E Moore, Philip F Stahel

**Affiliations:** 1 Department of Orthopaedic Surgery, Denver Health Medical Center, University of Colorado School of Medicine, Denver, CO 80204, USA; 2 Department of Trauma and Reconstructive Surgery, Charité University Medical Center, Campus Benjamin Franklin, 12200 Berlin, Germany; 3 Department of Surgery, Denver Health Medical Center, University of Colorado School of Medicine, Denver, CO 80204, USA

## Abstract

Major trauma induces marked metabolic changes which contribute to the systemic immune suppression in severely injured patients and increase the risk of infection and posttraumatic organ failure. The hypercatabolic state of polytrauma patients must be recognized early and treated by an appropriate nutritional management in order to avoid late complications. Clinical studies in recent years have supported the concept of "immunonutrition" for severely injured patients, which takes into account the supplementation of Ω-3 fatty acids and essential aminoacids, such as glutamine. Yet many aspects of the nutritional strategies for polytrauma patients remain controversial, including the exact timing, caloric and protein amount of nutrition, choice of enteral versus parenteral route, and duration. The present review will provide an outline of the pathophysiological metabolic changes after major trauma that endorse the current basis for early immunonutrition of polytrauma patients.

## Introduction

Severe trauma induces massive changes of the physiological state by alteration of metabolic pathways and activation of the innate immune system [1-5]. The posttraumatic metabolic changes are characterized by hypermetabolism with increased energy expenditure, enhanced protein catabolism, insulin resistance associated with hyperglycemia, failure to tolerate glucose load, and high plasma insulin levels ("traumatic diabetes") [1,2,6-11]. The alterations of the physiological metabolic pathways leads to the development of hyperglycemia and metabolic acidosis with hyperlactatemia [10,12]. The increased oxygen demands of the polytraumatized patient further aggravate the hypermetabolic state by enhanced mitochondrial oxygen utilization [1,10,13,14].

Metabolic changes after trauma were described more than six decades ago by Cuthbertson (*Lancet *1942, 1:433–437) and characterized as occurring in two different phases, termed the *"ebb" phase *and the *"flow" phase *(table 1). The *"ebb" phase *is initiated within minutes after trauma and persists for several hours after the initial insult. It is characterized by a decline in body temperature and oxygen consumption, aimed at reducing posttraumatic energy depletion. However, the brief duration of this phase limits its clinical relevance. The *"flow" phase*, which occurs after compensation of the state of traumatic-hemorrhagic shock, is associated with an increased metabolic turnover, activation of the innate immune system and induction of the hepatic acute-phase response [2,3]. This results in an increase of the catabolic state with a significantly increased consumption of energy and oxygen [2,10,13,14]. The amount of oxygen consumption and demand in patients with traumatic-hemorrhagic shock can be calculated using a formula described by Nunn and Freeman in 1964 (table 2) [15].

**Table 1:** Metabolic changes after major trauma.

***"Ebb" *phase (hours)**	***"Flow" *phase (days to weeks)**
Decreased body temperature	Increased body temperature
Decreased oxygen consumption	Increased oxygen consumption
Lactate acidosis	Negative nitrogen balance
Increased stress hormone levels	Increased stress hormone levels
Decreased insulin levels	Normal to increased insulin levels
Hyperglycaemia, insulin resistance	Hyperglycaemia, insulin resistance
Gluconeogenesis	Gluconeogenesis
Increased substrate consumption	Proteinolysis ("autocannibalism")
Hepatic acute-phase response	Lipolysis
Immune activation	Immunosuppression

**Table 2:** Calculation of available oxygen (O_2_*av *in ml/min) in bleeding polytrauma patients according to the formula described by Nunn and Freeman in 1964 [15].

**O_2_*av *= CO × S_a_O_2 _× Hb × 1.34**

[CO, cardiac output (ml/min); S_a_O_2_, arterial oxygen saturation (%); Hb, hemoglobin concentration (g%); 1.34, O_2 _binding capacity constant (ml O_2_/g Hb)].

In addition to the acute hypermetabolic state, the systemic inflammatory cascade is initiated as a consequence of trauma, as characterized by the release of pro-inflammatory cytokines and activation of the complement system [4,5,16,17]. The bacterial translocation caused by the traumatic-hemorrhagic shock may further aggravate these metabolic sequelae and inflammatory response [5,10,17-19], but this issue remains controversial [20]. Additionally, the frequent use of vasoactive drug therapy for hemodynamic resuscitation in traumatic-hemorrhagic shock has a profound impact on metabolism and organ energy status of the injured patient [1,10]. Most severely injured patients require inotropic support to promote hemodynamic stability. For example dopamine, a commonly used epinephrine precursor, leads to depression of pituitary function and inhibition of prolactin and growth hormone production [21]. Thus, the use of vasoactive drugs further promotes catabolism by reducing serum levels of anabolic hormones. In contrast, endogenous catecholamines, cortisol and glucagon levels are highly elevated after trauma, leading to increased energy substrate mobilization [3,6]. Interestingly, studies in severe burn patients have shown that exogenous insulin administration can attentuate protein catabolism as indicated by an increase in protein synthesis [11,22,23]. Proteinolysis of skeletal muscle and glycolysis are increased with the aim to provide the substrates for the hepatic gluconeogenesis and the hepatic biosynthesis of acute-phase proteins [2,24]. The metabolic state is reoriented towards supporting the organism's immune response and wound healing at the cost of enhanced proteinolysis of skeletal muscle [1,2]. In addition, the physical and psychological stimulation of the neuroendocrine axis through fear, stress, pain, inflammation and shock increases the caloric turnover significantly above the baseline situation in healthy individuals [6,10]. This leads to increased serum levels of catabolic hormones, such as cortisol, glucagon and catecholamines, and decreased levels of insulin causing the posttraumatic catabolic diabetic phase [10,11,25]. In contrast, the phenomenon of "occult adrenal insufficiency" has been demonstrated to occur in severely injured patients in the ICU, as defined by a serum cortisol below 18 mg/dL [26] or below 25 mg/dL [27] in different publications. However, up to present the clinical implication of posttraumatic adrenal failure with regard to patient outcome remains controversial [26-29].

Depending on the severity of the initial injury and the quality of the therapeutic regimen for the polytraumatized patients, catabolic changes in posttraumatic metabolism can last for several days or weeks [3].

## Metabolic control and immunonutrition

The state of hypercatabolism after severe injury can lead to severe complications associated with posttraumatic hyperglycemia, hypoproteinemia, lactate acidosis, and immunosuppression [2,8,10]. Thus, the presence and significance of these metabolic alterations must be recognized and appreciated in severely injured patients. An optimal therapeutic regimen should include the concept of a "metabolic control" in addition to the initial measures of resuscitation by hemorrhage control and securing airways and oxygenation [2,30,31]. The posttraumatic catabolic state requires an adjusted energetic balance with early protein substitution and hypercaloric nutrition [2,8,30,31]. Patients with major injuries who receive no nutrition during the first few days after trauma can develop cumulative caloric and protein deficits which contribute to the risk of increased complications, such as infections and organ failure [2,8,32]. Consequently, clinical pathways and algorithms for nutritional support of severely injured patients during the intensive care period have been developed in recent years [8,10,12,30,31,33]. These protocols are designed to restore the capacity for optimal immune and inflammatory responses and to facilitate the recovery and healing from trauma and subsequent infections. Early enteral nutrition has been advocated as the concept of choice for nutrition of polytraumatized and severely ill patients. In this regard, prospective randomized controlled trials have clearly demonstrated the positive effect of an early full enteral nutrition with a decreased posttraumatic infection rate, a shorter duration of hospital stay, and an improved overall outcome [32,34-42].

The specialized nutritional support for severely injured patients includes the administration of "immune nutrient cocktails" which have been shown to improve the survival of septic patients during the intensive care period [33,43]. The concept of "immunonutrition" has been established in recent years and exemplified by the enteral supplementation of glutamine, one of the most promising new nutritional concepts for severely injured patients in recent years [32,37,44-47]. Glutamine is an essential aminoacid which exerts metabolic benefits beyond its nutritional value by mediating immunological effects, such as induction of neutrophil phagocytic activity and oxidative burst [32,48,49]. Glutamine was also shown to protect neutrophils from undergoing apoptosis *in vivo *[50]. In addition, glutamine is a precursor to the reducing agent glutathione and thus contributes to antioxidant effects and cellular protection from ischemia/reperfusion-mediated injury [45,51]. This protective effect of glutamine has been demonstrated in different experimental models of ischemia/reperfusion injury [52,53]. Also, models of experimental starvation have shown the important nutritional effect of glutamine for enterocytes and intestinal mucosa [8]. Furthermore, glutamine has been shown to restore cellular energy reserves to normal levels after hemorrhagic shock and to attenuate the extent of shock-induced cellular apoptosis [54]. This finding is supported by reduced bacterial translocation in rat guts and improved gut immune function after diet supplementation with glutamine [45]. A prospective, randomized, double-blind controlled clinical trial demonstrated that glutamine supplementation reduces the incidence of multiple organ failure and death attributed to infections in critically ill patients [32]. In addition to glutamine, Ω-3 fatty acids have become an important nutritional supplementation for severely injured patients in recent years [55-57]. These long-chain polyunsaturated fatty acids derived from fish oil were shown to exert potent anti-inflammatory properties in trauma patients, such as attenuation of arachidonic acid-derived metabolites like prostaglandin PGE_2 _and leukotriene LTB_4_, inhibition of leukocyte activation and chemotaxis, and attenuation of pro-inflammatory gene expression levels [39,57-59].

Other nutritional supplements that promote anabolism in trauma patients include phospholipids, leptins, and anabolic hormones, such as thyroid hormones, growth hormone, and insulin [8,22,23,60,61]. For example, growth hormone substitution has been shown to promote protein anabolism in severely injured patients [62].

Recently published consensus guidelines based on meta-analyses from multiple prospective trials have helped clarify the indication for supplementation of specific nutrients in the clinical setting [31,44,63-65]. Moreover, the use of vasoactive therapy has been shown to influence not only the systemic and regional perfusion and organ blood flow, but also to affect the balance between oxygen and substrate supply [66]. The use and necessity of exogenous catecholamines should therefore be well calculated and balanced against the potential adverse influence on metabolic needs during the catabolic phase of critically ill trauma patients. Furthermore, there is evolving evidence that over-resuscitation, i.e. driving oxygen delivery to supraphysiological levels, may be deleterious [67].

## Clinical implications for severely injured patients

Although several studies have shown the positive effect of immunonutrition, its clinical impact remains debated, and the benefit may be specific to certain patient populations [68-70]. Despite this lack of consensus, the concept of early enteral feeding is generally accepted among trauma surgeons and intensive care physicians [32,34-42]. More than a decade ago, Moore and colleagues demonstrated in prospective randomized trials on patients with abdominal injuries [71] the positive effect of early enteral nutrition with a significant reduction of intraabdominal and pulmonary infections [34-36,72]. When compared to total parenteral nutrition, the enteral route is associated with a significantly decreased incidence of septic complications [39]. Although anecdotal reports have been published on the adverse effects and complications of enteral feeding procedures in critically ill patients [73], the overall consensus in the literature clearly advocates for the positive effects of early enteral nutrition in severely injured patients [31,42,43,68,72,74,75]. An issue under debate in clinical practice is the question of when early immunonutrition should be initiated [65]. This question has not been fully adressed in the current literature and requires further analysis with future clinical trials [76].

In daily clinical practice, the individual caloric requirement for severely injured patients should be assessed with defined algorithms, tables and equations. For example, the method of indirect calorimetry by the Weir equation helps to assess the energy expenditure by the parameters oxygen consumption and carbon dioxide production [7,77]. The basic metabolic rate can be calculated by the Harris-Benedict equation using the standard variables of height, weight, age and gender [7,78]. However, it is of key importance not to "overfeed" critically injured patients with calories, since this may contribute to adverse outcome [1,39,79,80]. Early overfeeding of severely injured patients leads to an increase in overall oxygen consumption, carbon dioxide production, hepatic lipogenesis, and hyperglycemia, and thus contributes to secondary immune suppression during the ICU phase [80]. Obese patients are particularly susceptible to the adverse effects of overfeeding. Therefore, a hypocaloric (<20 kcal/kg/day), high-protein nutrition was postulated as a safe protocol for critically injured obese patients and shown to be as effective as eucaloric or hypercaloric feeding (>20 kcal/kg/day) in this specific patient group [81-83]. Current feeding recommendations for morbidly obese ICU patients are about 20 kcal and 2 g of protein per kg ideal body weight per day [83].

The standard daily doses of protein, glucose, fat and amino acid concentrations must be clearly defined and adjusted to the calculated individual patients metabolic needs [77-80,83,84]. An example of a standardized enteral nutrition is Impact^®^; one of the most commonly used clinical formulas. Multicenter prospective randomized clinical trials on critically ill trauma patients have demonstrated that the administration of Impact^® ^for 7 to 10 days reduced the rates of infection, wound complications, and the risk of multiple organ failure [36,72,75]. Nonetheless, standardized enteral nutrition is not consistently administered to critically ill trauma patients. International clinical guidelines for nutritional support in critically ill patients have been published by the *American Society of Parenteral and Enteral Nutrition (ASPEN)*, the *Canadian Society for Clinical Nutrition *and more recently the *European Society of Parenteral and Enteral Nutrition (ESPEN) *[76,85,86].

**Figure 1:**
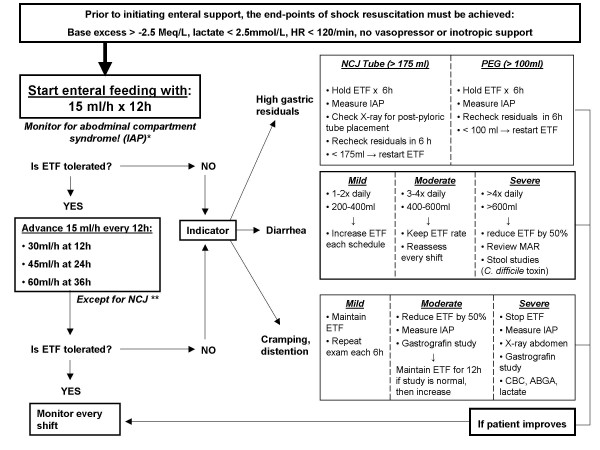
**Denver Health Medical Center institutional protocol for early enteral nutrition of severely injured patients**. Adapted from: [39]. Abbreviations: ABGA, arterial blood gas analysis; ATI, Abdominal Trauma Index; CBC, complete blood count; HR, heart rate; ETF, enteral tube feeding; IAP, intraabdominal pressure (bladder pressure); ISS, Injury Severity Score; MAR, medicine administration record; NCJ, needle catheter jejunostomy; PEG, percutaneous endoscopic gastrostomy; PRBC, packed red blood cells. * *Monitoring of IAP for high risk patients with severe pelvic ring injuries, lumbar spine fractures, polytrauma with ISS > 17, hemorrhagic shock with > 6 units PRBC in 12 h*. ** *In massively injured patients (ISS>40, ATI>40, PRBC mass transfusions), administer low dose enteral feeding (15–30 ml/h) for the first 3 days due to anticipated intolerance to full-dose enteral feeding. Advance per protocol on the 4^th ^day post injury*.

A review by Wernerman provides a detailed analysis of the weaknesses and strengths of the respective recommendations for nutritional support of critically ill patients [87]. Altogether, the guidelines clearly favor the concept of early enteral nutrition within 24–48 hours after admission in the intensive care unit [87]. For daily use in clinical practice, the reader is referred to the published clinical guidelines for nutritional support [39,76,85,86]. The main recommendations derived from the ESPEN guidelines are summarized in table 3. Furthermore, our own institutional protocol for nutritional support of severely injured patients is shown in figure 1. The clinical value of these guidelines is awaiting scientific validation by implementation in future prospective randomized trials.

**Table 3:** Summary of the main recommendations from the ESPEN guidelines for enteral nutrition of critically ill patients. Adapted from: [76].

	**Recommendations**
**Indications and application of enteral nutrition (EN)**	All patients who are not expected to be on a full oral diet within three days.
	The expert committee recommends that haemodynamically stable critically ill patients who have a functioning gastrointestinal tract should be fed early (<24 h) using an appropriate amount of nutrition.
	Exogenous energy supply (kcal):
	• 20–25 kcal/kg body weight/day during the acute and initial phase of critical illness.
	• 25–30 kcal/kg body weight/day during the anabolic recovery phase,
	Consider parenteral administration of metoclopramide or erythromycin in patients with intolerance to enteral feeding (e.g. with high gastric residuals).
**Route of administration**	Use EN in all patients who can be fed via the enteral route.
	There is no significant difference in the efficacy of jejunal versus gastric feeding in critically ill patients.
	Avoid additional parenteral nutrition in patients who tolerate EN and can be fed to the target values.
	Consider careful parenteral nutrition in patients intolerant to EN.
**Type of formula**	Whole protein formulae are appropriate in most patients, since peptide-based formulae have not shown clinical advantages.
	"Immunonutrition":
	Glutamine should be added to standard enteral formula in all trauma patients and burn patients.
	Formulae enriched with nucleotides and fatty acids are superior to standard enteral formulae in trauma patients, patients with ARDS, and patients with mild, but not severe, sepsis (APACHE II score < 15)
	Patients with very severe illness who do not tolerate more than 700 ml enteral formulae per day should not receive an immune-modulating formula.

## Conclusion

Dramatic metabolic changes occur in severely injured patients which must be acknowledged early and monitored during the posttraumatic phase. Appropriate immunonutrition should be started in the ICU, preferably by enteral route, in order to counteract the potentially devastating effects of the massive hypermetabolic state after major trauma. Recently published international guidelines on enteral nutrition concepts in critically ill patients are available for implementation in clinical practice and future prospective studies.

## Competing interests

None.

## Authors' contributions

EH, AW, IL, SJM, WRS, EEM, and PFS contributed equally to the conception and design, literature appraisal and writing of this manuscript. The final version of this paper was approved by all authors.
